# Functional outcomes in the inpatient rehabilitation setting following severe COVID-19 infection

**DOI:** 10.1371/journal.pone.0248824

**Published:** 2021-03-31

**Authors:** Cameron Spencer Olezene, Elizabeth Hansen, Hannah K. Steere, Joseph T. Giacino, Ginger R. Polich, Joanne Borg-Stein, Ross D. Zafonte, Jeffrey C. Schneider

**Affiliations:** Department of Physical Medicine and Rehabilitation, Spaulding Rehabilitation Hospital, Harvard Medical School, Charlestown, Massachusetts, United States of America; Ohio State University Wexner Medical Center Department of Surgery, UNITED STATES

## Abstract

**Objective:**

To characterize the functional impairments of a cohort of patients undergoing inpatient rehabilitation after surviving severe COVID-19 illness, in order to better understand the ongoing needs of this patient population.

**Methods:**

This study consisted of a retrospective chart review of consecutive patients hospitalized for COVID-19 and admitted to a regional inpatient rehabilitation hospital from April 29^th^ to May 22^nd^, 2020. Patient demographics, clinical characteristics and complications from acute hospitalization were examined. Measures of fall risk (Berg Balance Scale), endurance (6 Minute Walk Test), gait speed (10 Meter Walk Test), mobility (transfer and ambulation independence), cognition, speech and swallowing (American Speech and Hearing Association National Outcomes Measurement System Functional Communication Measures) were assessed at rehabilitation admission and discharge.

**Results:**

The study population included 29 patients and was 70% male, 58.6% white and with a mean age of 59.5. The mean length of acute hospitalization was 32.2 days with a mean of 18.7 days intubated. Patients spent a mean of 16.7 days in inpatient rehabilitation and 90% were discharged home. Patients demonstrated significant improvement from admission to discharge in measures of fall risk, endurance, gait speed, mobility, cognition, speech and swallowing, (p< 0.05). At discharge, a significant portion of the population continued to deficits in cognition (attention 37%; memory 28%; problem solving 28%), balance (55%) and gait speed (97%).

**Conclusion:**

Patients admitted to inpatient rehabilitation after hospitalization with COVID-19 demonstrated deficits in mobility, cognition, speech and swallowing at admission and improved significantly in all of these domains by discharge. However, a significant number of patients exhibited residual deficits at discharge highlighting the post-acute care needs of this patient population.

## Introduction

Severe Acute Respiratory Syndrome Coronavirus-2 (SARS-CoV-2) is a novel coronavirus that causes coronavirus disease 2019 (COVID-19) [1]. The spread of COVID-19 has caused a global pandemic resulting in significant morbidity and mortality worldwide. COVID-19 most commonly causes upper respiratory symptoms, but can progress to lower respiratory infection, resulting in acute respiratory distress syndrome and acute respiratory failure [2]. COVID-19 infection has also been shown to cause many different complications including hypercoagulability [3], stroke [4], myocarditis [5], acute coronary syndrome [6] and liver injury [7].

Patients that survive severe COVID-19 illness develop a myriad of functional deficits that impact their ability to return home from the acute care hospital. Cardiopulmonary symptoms include reduced aerobic capacity, orthostatic hypotension and arrhythmias [5, 8, 9]. This population also demonstrates impaired balance, strength and sensation [10]. Neurologic sequelae have included meningitis, encephalitis, critical illness polyneuropathy and stroke [8, 11]. Additionally, cognitive deficits have been noted in areas of memory, attention, problem solving and protracted delirium [8]. Furthermore, patients commonly develop dysphagia from prolonged intubation necessitating a gastrostomy tube [9].

There is not much data on the rehabilitation course and functional outcomes following prolonged hospitalization for COVID-19. It has been shown that a large proportion of patients have persistent symptoms that impact quality of life [12]. Patients with delirium are more likely to develop deficits in cognition and activities of daily living function [13]. Additionally, patients with strokes and COVID-19 have worse function on the modified Rankin scale than stroke patients without COVID infection [14]. Patients exhibit significant improvement in pulmonary function after weeks of outpatient respiratory therapy [15]. However, inpatient rehabilitation functional data in the COVID-19 survivor population is limited. Given the global burden of COVID-19 disease and the increasing need for rehabilitation in this population, it is important to understand the multidimensional functional impairments and extent of functional improvement achieved by this population during inpatient rehabilitation.

## Materials and methods

The study consisted of a convenience sample of consecutively admitted patients to an inpatient rehabilitation facility (IRF) in Boston, MA, following hospitalization for COVID-19 between April 29^th^ and May 22^nd^, 2020. Demographic, clinical and outcomes data were collected by retrospective chart review using a standardized data extraction form.

All subjects received standard of care inpatient rehabilitation. Rehabilitation therapy treatments are performed by physical therapists, occupational therapists and speech and language pathologists at least three hours per day, five days per week. There was not a standardized treatment protocol for post-COVID patients. Interventions were tailored to each patient’s individual needs.

Functional measures were assessed at IRF admission and discharge. Outcomes assessed included fall risk (Berg Balance Scale (BBS)), endurance (6 Minute Walk Test (6MW)), gait speed (10 Meter Walk Test (10MW)), mobility (transfer and ambulation independence), as well as cognition, speech and swallowing (American Speech and Hearing Association National Outcomes Measurement System Functional Communication Measures (FCM)). The BBS is a validated 14 item assessment that evaluates static balance and risk of falls [16]. The 6MW evaluates endurance by measuring the distance a patient can walk in six minutes [17]. The 10MW evaluates gait speed by measuring how quickly a patient can walk 10 meters [18]. FCMs are scored on a seven-point ordinal scale in fifteen different domains [19]. An FCM domain was evaluated for a patient if they demonstrated deficits in that domain at IRF admission. The domains consistently assessed were voice, swallowing, attention, memory and problem solving. All measures have established validity. Wilcoxon Signed-Rank and Chi-Squared tests were used to assess differences between admission and discharge assessments for ordinal and categorical data, respectively. A p-value of less than 0.05 indicated significance. Clinically meaningful improvement from IRF admission to discharge for BBS [20], 6MW [21] and 10MW [22] was evaluated using established minimal detectable change (MDC). Persistent deficits at IRF discharge were defined as the proportion of patients with BBS less than 45, (indicating increased fall risk) [23], 10MW below age and gender normative values [24] and FCM domain scores less than the maximum of seven in each domain [19]. The institutional review board for Mass General Brigham approved this study as IRB exempt due to the retrospective nature of the study. Patient consent was not required due to the low risk associated with the anonymous reporting of data.

## Results

This study included 29 patients, who were 70% male, 58.6% white and had a mean age of 59.5 years. The most common preexisting medical conditions were hypertension (76%), obesity (62%) and hyperlipidemia (55%). All patients required intubation. The mean length of intubation was 18.7 days and acute hospital stay was 32.2 days. Dysphagia (86.2%), weight loss (79.3%) and delirium (69%) were the most common complications. Tracheostomy was performed in 20.7% of patients, while gastrostomy was performed in 13.8% of patients. Significant weight loss occurred in 79.3% of patients with a mean loss of 12.5% of premorbid body weight compared to weight at IRF admission. Pressure injuries occurred in 37.9% of patients, which included common locations from lying supine (sacrum/ankles) and uncommon location from lying prone on the ventilator (face/abdomen) (Table 1).

**Table 1:** Demographic and clinical characteristics of the study sample (n = 29).

	Number of participants (percentage)
**Male sex, No. (%)**	20 (70%)
**Age, median years (IQR)**	60 (50.5–67.5)
**Race/ethnicity, No. (%)**	
White	17 (58.6%)
Hispanic not otherwise specified	6 (27.6%)
Asian	4 (13.8%)
Black/African American	2 (6.9%)
**Preexisting medical conditions, No. (%)**	
Hypertension	22 (75.9%)
Obesity	18 (62.1%)
Hyperlipidemia	16 (55.2%)
History of Smoking ^a^	14 (48.2%)
Diabetes mellitus type 2	11 (37.9%)
Coronary artery disease	5 (17.2%)
Chronic kidney disease	5 (17.2%)
Obstructive sleep apnea	4 (13.8%)
Asthma	2 (6.9%)
Congestive heart failure	1 (3.4%)
Chronic obstructive pulmonary disease	1 (3.4%)
**Intubation, No, (%)**	29 (100%)
**Length of intubation mean days (SD)** ^b^	18.7 (5.7)
**Length of hospitalization, mean days (SD)**	32.2 (9.3)
**Medical complications**	
Dysphagia	25 (86.2%)
Weight loss	23 (79.3%)
Delirium	20 (69.0%)
Acute kidney injury	17 (58.6%)
Hospital acquired pneumonia	15 (51.7%)
Acute liver injury	13 (44.8%)
Hypercoagulability ^c^	9 (31%)
Supine pressure injury ^d^	8 (27.6%)
Tracheostomy	6 (20.7%)
New onset atrial fibrillation	5 (17.2%)
Critical illness myopathy	7 (24.1%)
Critical Illness neuropathy	3 (10.3%)
Gastrostomy tube	4 (13.8%)
Prone pressure injury ^e^	4 (13.8%)
Stroke	3 (10.3%)
Deep vein thrombosis	2 (6.9%)
Acute coronary syndrome	1 (3.4%)
ECMO	1 (3.4%)
**Length of inpatient rehabilitation, mean days (SD)**	16.7 (7.8)
**Inpatient rehabilitation disposition, No. (%)**	
Home	26 (90.0%)
Skilled nursing facility	0 (0%)
Planned readmission	2 (7.1%)
Unplanned readmission	1 (3.6%)
**Deficits noted during rehabilitation, No. (%)**	
Diffuse Weakness ^f^	15 (51.7%)
Focal Weakness ^g^	4 (13.8%)
Sensory Loss	6 (20.7%)
Hand tremors	11 (37.9%)
Sinus tachycardia	10 (34.5%
Orthostatic hypotension	7 (24.1%)
Vertigo	3 (10.3%)

Abbreviations: IQR, interquartile range; SD, Standard Deviation; ECMO, extracorporeal membrane oxygenation

^a^ Includes active and former smokers

^b^ Excludes patients that required tracheostomy

^c^ Patients documented as having abnormal clotting of access catheters or presence of deep venous thrombosis

^d^ Includes wounds on sacrum, ischial tuberosities, ankles, heels and elbows

^e^ Includes wounds on face and abdomen

^f^ Medical Research Council (MRC) grade less than 5 involving all limbs symmetrically

^g^ MRC grade less than 5 in one or more limbs asymmetrically

The mean length of inpatient rehabilitation was 16.7 days. Most patients (90%) were discharged home. No patients were discharged to a skilled nursing facility. Two patients had planned readmissions for surgical treatment of pressure injuries. One patient had an unplanned admission for a hospital acquired pneumonia (Table 1).

Table 2 presents functional outcomes at IRF admission and discharge. The population demonstrated deficits in all domains examined at admission. Patients demonstrated statistically significant improvements on the BBS (p<.001), 10MW (p<.001), 6MW (p<.001), transfer independence (p<.001), ambulation independence (p<.001) and all FCM domains (voice (p<.05), swallowing (p<.001), attention (p<.001), memory (p<.001), problem solving (p<.001)). Minimal detectable change from admission to discharge was demonstrated in 75% of subjects for BBS, 94% for 10MW and 100% for 6MW. However, many patients demonstrated persistent deficits in cognition (attention 37%; memory 28%; problem solving 28%) at discharge. They also had persistent deficits in balance (55% with BBS <45 indicating greater fall risk) and gait speed (97% with 10MW below age/gender norms).

**Table 2:** Comparison of inpatient rehabilitation functional measures at admission and discharge.

Outcome Measure	Admission Assessment	Discharge Assessment	p-value ^a^
Berg Balance Scale, mean (SD), (n = 24)	22.6 (18.5)	43.7 (14.0)	<0.001*
10 Meter Walk Test, mean meters per second (SD), (n = 17)	0.25 (0.25)	0.86 (0.57)	<0.001*
6 Minute Walk Test, mean meters (SD), (n = 19)	206.6 (258)	764.5 (276.1)	<0.001*
Functional Independence, No. (%)			
Transfer independence (n = 29)	1 (3.4%)	27 (93.1%)	<0.001*
Ambulation independence (n = 29)	0 (0%)	25 (86.2%)	<0.001*
Functional Communication Measure, median (IQR)			
Voice (n = 6)	4 (4–5)	6.5 (4.75–7)	0.032*
Swallowing (n = 18)	4 (3–5)	7 (7–7)	<0.001*
Attention (n = 19)	4 (4–5)	7 (6–7)	<0.001*
Memory (n = 18)	4 (4–5)	7 (6.25–7)	<0.001*
Problem Solving (n = 18)	4 (4–5)	7 (6.25–7)	<0.001*

Abbreviations: IQR, Interquartile Range; SD, Standard Deviation; FCM, Functional Communication Measures; BBS, Berg Balance Scale; 10MW, 10 Meter Walk test; 6MW, 6 Minute Walk test

^a^ Differences between admission and discharge assessments were evaluated with Wilcoxon Signed-Rank Test (BBS, 10MW, 6MW, FCM) and Chi-Squared test (Functional Independence)

*indicates statistical significance

Minimal detectable change is 6.3 for BBS, 0.05 for 10MW and 58 for 6MW. Minimal detectable change from admission to discharge was demonstrated in 75% of subjects for BBS, 94% for 10MW and 100% for 6MW.

BBS score <45, indicating a greater fall risk, was present in 16 (55.2%) patients at discharge.

Age and gender matched normative values for 10MW were not attained in 28 (96.5%) patients at discharge.

Maximum FCM score (independence) was documented in 3 (50%) for Voice, 14 (78%) for Swallowing, 12 (63%) for Attention, 13 (72%) for Memory and 13 (72%) for Problem Solving at discharge.

## Discussion

This study describes the clinical characteristics of a cohort of patients who underwent inpatient rehabilitation following hospitalization with severe COVID-19. Multidimensional functional deficits in mobility, cognition, speech and swallowing were pervasive at the time of admission to rehabilitation. Although the study population demonstrated significant improvements in all domains examined, deficits remained in domains of fall risk, gait speed and cognition at rehab discharge. This study highlights the prevalence of persistent functional deficits after severe COVID-19 that will require ongoing treatment and may, in some cases lead to longer-term impairments.

Information on the long-term effects of COVID-19 infection is limited. At approximately two months after onset of symptoms, fatigue and dyspnea are common [12]. Complications and functional consequences of prolonged hospitalization in the intensive care unit have elsewhere been referred to as Post-Intensive Care Syndrome (PICS) [11]. PICS is characterized by weakness, deconditioning, cognitive dysfunction and psychiatric illness that persists following resolution of acute illness [25]. Cognitive impairment affecting attention, concentration, memory and executive function often persists for years in post-ICU syndrome [26]. While the spectrum of complications that impact recovery from COVID-19 are not completely understood, there is likely overlap with PICS [10, 27].

Similar to sepsis survivorship initiatives, a large-scale registry to facilitate trials and detailed longitudinal follow-up is needed to advance understanding of COVID-19 recovery [28]. Of note, no patients were discharged to skilled nursing facilities. This may reflect concerns about COVID-19 transmission within facilities [29], and will likely change as the healthcare system adapts to the growing needs of COVID-19 survivors.

Study limitations include small sample size, single center, retrospective design and lack of standardized rehabilitation protocol for COVID-19 patients. Baseline functional status and psychiatric complications were not evaluated in this study. Despite these limitations, this study begins to illuminate the multidimensional functional impairments and post-acute care needs of this population.

## Supporting information

S1 Data (XLSX)
